# Clinical Impact of ^18^F-FDG PET/CT in the Diagnostic Workup of Pancreatic Ductal Adenocarcinoma: A Systematic Review

**DOI:** 10.3390/diagnostics10121042

**Published:** 2020-12-03

**Authors:** Annachiara Arnone, Riccardo Laudicella, Federico Caobelli, Priscilla Guglielmo, Marianna Spallino, Elisabetta Abenavoli, Anna Lisa Martini, Rossella Filice, Alessio Danilo Comis, Marco Cuzzocrea, Flavia Linguanti, Laura Evangelista, Pierpaolo Alongi

**Affiliations:** 1 Nuclear Medicine Unit, Department of Experimental and Clinical Biomedical Sciences “Mario Serio”, University of Florence, 50134 Florence, Italy; elisabettabenavoli@gmail.com (E.A.); alm051084@gmail.com (A.L.M.); flavialinguanti@hotmail.it (F.L.); 2 Department of Biomedical and Dental Sciences and of Morpho-Functional Imaging, Nuclear Medicine Unit, University of Messina, 98125 Messina, Italy; riclaudi@hotmail.it (R.L.); filicer@libero.it (R.F.); alessiodanilo.comis@gmail.com (A.D.C.); 3 Clinic of Radiology & Nuclear Medicine, University Hospital Basel, University of Basel, 4031 Basel, Switzerland; federico.caobelli@gmail.com; 4 Nuclear Medicine Division, University Hospital of Parma, 43126 Parma, Italy; priscilla.guglielmo@yahoo.it; 5 Nuclear Medicine Unit, ASST “Papa Giovanni XXIII”, 24127 Bergamo, Italy; mariannaspallino@gmail.com; 6 Nuclear Medicine, Fondazione IRCCS Ca’ Granda Ospedale Maggiore Policlinico, 20122 Milan, Italy; marco.cuzzocrea@policlinico.mi.it; 7 Nuclear Medicine Unit, Department of Medicine, Padova University Hospital, Via Giustiniani 2, 35128 Padova, Italy; laura.evangelista@unipd.it; 8 Unit of Nuclear Medicine, Fondazione Istituto G.Giglio, 90015 Cefalù, Italy; alongi.pierpaolo@gmail.com

**Keywords:** pancreas, positron emission tomography, PET/CT, pancreatic ductal adenocarcinoma, FDG

## Abstract

In this review, the performance of fluorodeoxyglucose (FDG)-positron emission tomography (PET)/computed tomography (CT) in the diagnostic workup of pancreatic ductal adenocarcinoma (PDAC) is evaluated. A comprehensive literature search up to September 2020 was performed, selecting studies with the presence of: sample size ≥10 patients and index test (i.e., “FDG” or “18F-FDG” AND “pancreatic adenocarcinoma” or “pancreas cancer” AND “PET” or “positron emission tomography”). The methodological quality was evaluated using the revised quality assessment of diagnostic accuracy studies (QUADAS-2) tool and presented according to the preferred reporting items for systematic reviews and meta-analyses (PRISMA) guidelines. Basic data (authors, year of publication, country and study design), patients’ characteristics (number of enrolled subjects and age), disease phase, type of treatment and grading were retrieved. Forty-six articles met the adopted research criteria. The articles were divided according to the considered clinical context. Namely, besides conventional anatomical imaging, such as computed tomography (CT) and magnetic resonance imaging (MRI), molecular imaging with FDG PET/CT is an important tool in PDAC, for all disease stages. Further prospective studies will be necessary to confirm the cost-effectiveness of such imaging techniques by testing its real potential improvement in the clinical management of PDAC.

## 1. Introduction

Pancreatic ductal adenocarcinoma (PDAC) represents a very aggressive and lethal cancer characterized by high mortality and short survival time [1]. Globally, pancreatic cancer is the 7th leading cause of cancer deaths, causing more than 330,000 deaths per year. PDAC is the 12th most common cancer worldwide with the highest incidence in men in North America and Europe [1] and Cancer statistics 2020 reported that the five-year survival rate is only 9% [2]. The poor prognosis of PDAC is due to its highly aggressive nature and its propensity for early dissemination to regional and distant sites [3]. Radical surgical procedures potentially allow the curing of patients with PDAC but less than 20% of them have a localized cancer at diagnosis [4,5], due to the lack of a useful screening biomarker [6,7] and to the non-specific and late nature of PDAC symptoms [3]. The remaining patients (stage III, 35% and stage IV, 50%) have inoperable PDAC and are usually treated with combined therapies [8], with a five-year survival rate of only 2% [9]. Therefore, an early diagnosis and an accurate staging are of paramount clinical significance for determining the optimal initial management strategy [10] and for establishing predictors of clinical outcomes. Computed tomography (CT) is actually the main imaging modality to detect PDAC [11,12], representing the first-line tool for tumor staging and therapeutic decision making [7,13]. Conversely, the role of ^18^F-fluorodeoxyglucose (FDG) positron emission tomography/CT (PET/CT) in PDAC is currently not clear and it is still considered to be “under development” [7,8]. This paper aims to systematically review the existent literature about the clinical impact of FDG PET/CT in PDAC in different disease phases, such as diagnosis, preoperative staging, prognosis, tumor recurrence and treatment response assessment.

## 2. Materials and Methods

A comprehensive literature search in the Pubmed/Scopus/Google Scholar/Cochrane/EMBASE databases was performed to identify the most relevant studies published up to September 2020 about the role of FDG PET/CT in PDAC. Studies were selected through the Preferred Reporting Items for Systematic reviews and Meta-Analyses (PRISMA) PRISMA method, using the following keywords: “FDG” or “18F-FDG” AND “pancreatic adenocarcinoma” or “pancreas cancer” AND “PET” or “positron emission tomography”. Some filters were used, such as original articles, English language, no preclinical studies. Original articles with less than 10 patients, editorials, case reports and review articles were excluded. Four independent authors reviewed the retrieved full texts in order to verify the relevance of data and then divided the articles based on clinical setting (i.e., diagnosis, preoperative staging, prognosis, response to treatment assessment and change of management). For each included study general information was attained, such as basic data (authors, year of publication and study design), patients’ characteristics (number of enrolled subjects, mean or median age), disease phase, type of treatment and grading. Additionally, in the case of studies that included the same population, only the report with the highest number of enrolled patients was considered. This systematic review was carried out using established methods and the presentation of results was made according to the PRISMA guidelines [14].

### Quality Assessment

The overall quality of the included studies in the meta-analysis was evaluated using the revised QUality Assessment of Diagnostic Accuracy Studies (QUADAS-2) tool [15]. This method comprises four domains (patients’ selection, index test, reference standard and flow, and timing). Each domain was assessed considering risk of bias and the first three domains were assessed to confirm the applicability.

## 3. Results

**Figure 1:**
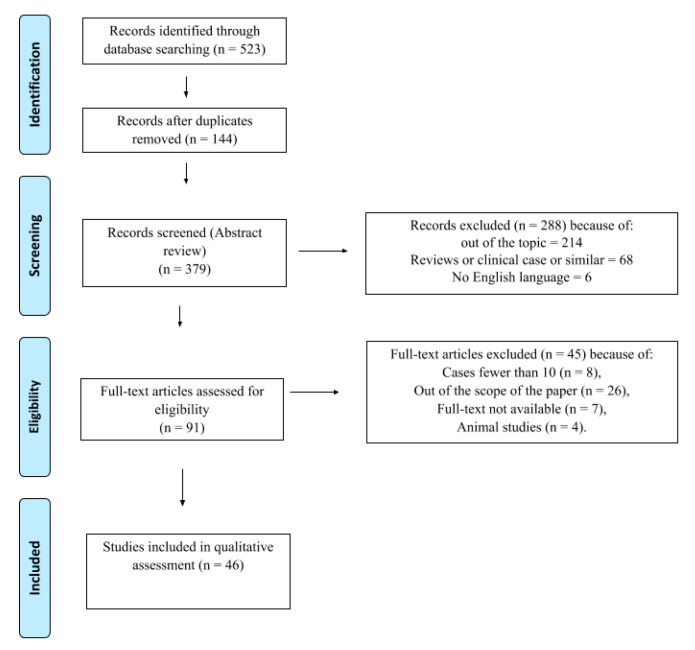
Preferred Reporting Items for Systematic reviews and Meta-Analyses (PRISMA) flow-chart. Selection process of studies included in the qualitative and quantitative analysis according to the PRISMA flow diagram [14].

A total of 523 articles were identified in the comprehensive literature search in Pubmed/Scopus/Google Scholar/Cochrane/EMBASE. Excluding duplicates, 379 articles were screened. Two-hundred eighty-eight articles were excluded (not associated with the main topic, reviews, clinical cases, no English language) and 91 articles were evaluated for eligibility. Forty-five articles out of 91 were deleted for the following reasons: cases fewer than 10 (n = 8), aim not within the scope of the paper (n = 26), full-text not available (n = 7), animal studies (n = 4). Forty-six studies were included in this systematic review (Figure 1) and were divided based on clinical setting (i.e., diagnosis, preoperative staging, prognosis, tumor recurrence, treatment response assessment and change of management; Table 1).

**Figure 2:**
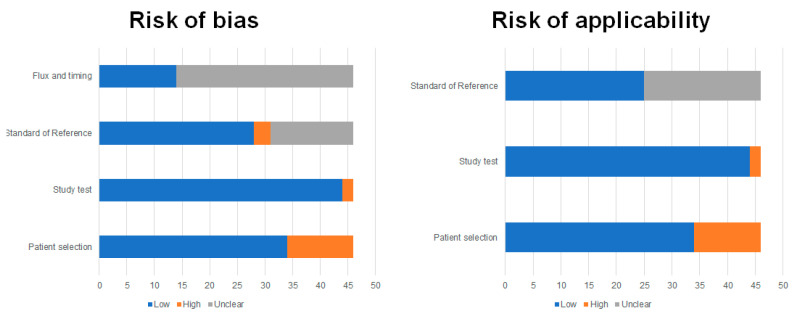
Study quality assessment. Overall quality assessment of the studies included in the systematic review according to the revised quality assessment of diagnostic accuracy studies (QUADAS-2) tool [15].

The Quadas-2 results are reported in Figure 2. As illustrated, many studies showed unclear flow and timing and, in some cases, no standard of reference was reported.

### 3.1. Diagnosis

FDG PET/CT imaging can improve the differential diagnosis between benign and malignant lesions based on the uptake of the radiopharmaceutical agent. Ergul et al. compared functional imaging with FDG PET/CT to conventional imaging such as Endoscopic Ultrasound (EUS), MRI and CT in the diagnosis of PDAC [16]. They assessed the equality in sensitivity and negative predictive value (NPV) between EUS and FDG PET/CT, resulting majorly compared to CT and MRI. Moreover, FDG PET/CT has proven to be superior to both specificity and positive predictive value (PPV) compared to CT. These findings were more effective in discriminating the benign and malignant lesions (with a sensitivity of 100% and a specificity of 89.5%) when a cut-off value of 3.2 was used for the maximum Semiquantitative Uptake Value (SUVmax). Therefore, FDG PET/CT seems to be useful, even more if combined with EUS, at the beginning of the diagnostic process [16].

Low specificity is one of the main limitations of PET because an increased FDG uptake may also be observed in inflammatory disease, thus significantly influencing the detection of primary tumor in the case of pancreatitis [62]. A significant association between FDG uptake and the size of the pancreatic tumor has also been reported. Patients in advanced tumor stages had a focally increased accumulation in PET images, while only a small percentage of patients in early stages showed increased FDG uptake [63].

Nowadays, PET does not appear as a diagnostic tool in some guidelines [7,8]. Nevertheless, in the study by Murakami et al. FDG PET/CT showed a high diagnostic performance in differentiating PDAC from pancreatitis because the uptake is homogeneous/solitary and heterogeneous/multifocal, respectively [62]. Additionally, Zhang et al. demonstrated that, especially when a biopsy is inconclusive, FDG PET/CT is helpful in the non-invasive diagnosis of autoimmune pancreatitis (AIP), particularly when using radiomics for the analysis of the images [17]. Similarly, Buchs et al. found that FDG PET/CT is useful for preoperative diagnosis in patients with suspected pancreatic cancer in whom CT alone failed to identify a small tumor or in whom fine needle aspiration (FNA) was not diagnostic [18]. Furthermore, it was seen that using an appropriate SUVmax cut-off of 1.5 [64] or 2.0 [65], or the retention index (RI) on dual-phase FDG PET/CT [65], the differential diagnosis can be easily made. However, this latter information is not completely considered true by Kato et al. who did not identify any cut-off value able to clearly divide AIP from PDAC [19]. Hu et al. also evaluated the role of SUVmax concerning the differential diagnosis between benign and malignant lesions and explored a possible correlation with the Ki67 index [20]. They found that SUVmax of the primary lesion had a positive correlation with Ki67, being significantly higher in malignant than benign lesions (6.3 ± 2.4 vs. 2.9 ± 2.0; *p* value < 0.001). Moreover, at receiving operating curve (ROC) analysis, the best specificity (76.9%) and sensitivity (92.6%) were obtained using SUVmax cut-off value of 3.5 [20]. From many clinical studies, it is also noticed that hyperglycemia can decrease sensitivity in detecting pancreatic cancer, and this feature can compromise the correct diagnosis because up to 80% of patients present early hyperglycemia or diabetes mellitus [66]. Chung et al. using a SUVmax cut-off value equal to 4.0, showed a decrease in sensitivity in the diabetes mellitus (DM) group (49.3%) compared to the non-DM group (75.5%). The sensitivity in the DM group increases to become comparable to that of the non-DM group when the cut-off SUVmax value is reduced to 3.196, inevitably decreasing the specificity. Therefore, in light of the results of this study, FDG images should be closely considered in diabetic patients with PDAC [66].

**Figure 3:**
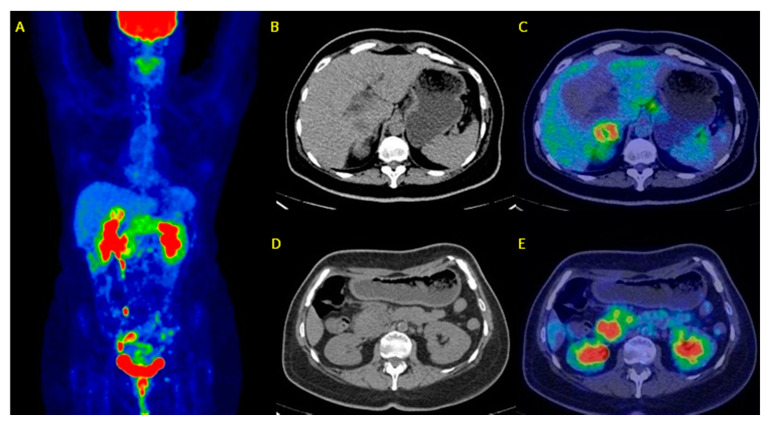
A sixty-two-year-old patient underwent ^18^F-FDG PET/CT for metabolic characterization of a pancreatic lesion. Whole Body FDG PET/CT (**A**: maximum intensity projection) revealed a focal uptake (SUVmax = 18) on the head of the pancreas (**D**,**E**) with a possible infiltration of the duodenum and a liver metastasis of S5 with a SUVmax = 12 (**B**,**C**). Histological samples of both uptakes confirmed the diagnosis of ductal adenocarcinoma infiltrating duodenum and liver metastasis. (Images from Nuclear Medicine Unit, Fondazione Istituto G.Giglio of Cefalù, Italy). Legend: **A** = Maximum intensity Projection (MIP); **B**-**D** = Axial images of low-dose CT; **C**-**E** = PET/CT fused images

In Figure 3 is reported an example of patients with suspected PDAC who underwent FDG PET/CT for a differential diagnosis.

### 3.2. Preoperative Staging

The aims of preoperative staging are to evaluate the resectability of the primary tumor and to detect distant metastases that would prevent surgery. In this context, contrast enhanced (ce) CT and MRI are the modalities of choice [13].

Myssayev et al. in a retrospective study of 48 patients, examined the operability of PDAC focusing on PET volumetric parameters, such as total lesion glycolysis (TLG) or metabolic tumor volume (MTV), and concluded that both are superior to SUV-based parameters for predicting tumor aggressivity [21]. However, both MTV and TLG are not correlated to vascular infiltration status and therefore cannot be helpful alone for taking decisions to perform surgery [21]. Strobel et al. in a 50-patients cohort, described the value of the one-stop-shop imaging approach, stressing the role of contrast-enhanced FDG PET/CT in defining the resectability of PDAC [22]. They assessed the following values concerning sensitivity and specificity, respectively: 100% and 44% for PET alone; 100% and 56% for unenhanced PET/CT; and 96% and 82% for enhanced PET/CT. All imaging techniques missed unresectability in five patients, which was later proved intraoperatively. The combined imaging approach was superior to unenhanced PET/CT, improving the detection of distant metastasis, with a sensitivity of 82% vs. 46% in liver metastasis and 80% vs. 60% in peritoneal carcinomatosis [22]. Similar results were revealed by Asagi et al. who found that enhanced PET/CT was significantly accurate concerning local invasion and distant metastasis [23], following Picchio et al. [24] and Casneuf et al. [25]. However, Lemke et al. showed that, in pancreatic cancer, the image fusion with PET/ceCT permits a more accurate assessment of the resection criteria, also improving the correct anatomic localization of small lesions [26]. Conversely, Yoneyama et al. in a pool of 95 patients, showed that the magnitude of diagnostic accuracy of contrast enhanced PET/CT in the detection of distant metastasis, lymph node metastasis and peritoneal dissemination remains still unclear [27]. Since lymph node metastasis is an independent negative prognostic factor and occurs frequently in PDAC, the possibility to evaluate the lymph node involvement before surgery or other treatments is also very relevant. Wang et al. showed that either CT or PET are limited in this aim (sensitivity about 40%) because several features, such as radioactive uptake or size, can lead to an altered evaluation of node metastasis [28]. They considered an SUVmax value of 7.05 in the primary tumor and Ca 19.9 value of 240.55 U/mL as the best thresholds to predict lymph node micrometastases, so helping in the selection of patients who can benefit from neoadjuvant treatment [28]. However, the SUV of the lymph nodes (SUVLN) seems to be a more significant prognostic factor in pancreatic cancer than the primary tumor’s SUV [29]. Finally, an important contribution of FDG PET has been reported by Kaida et al. concerning the correlation with molecular prognostic factors. Namely, the FDG uptake may predict the levels of the endothelial growth factor receptor (EGFR) and p70S6 expressions, whilst mTOR did not correlate with FDG uptake in the primary lesions [30].

**Figure 4:**
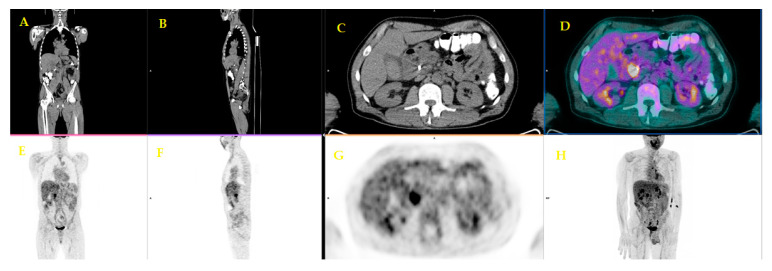
A fifty-six-year-old patient underwent staging FDG PET/CT with positive (ce)CT examination for local pancreatic lesion and equivocal for distant metastases. FDG PET/CT revealed a focal uptake on the head of the pancreas (**C**,**D**,**G**). (Images from Nuclear Medicine Unit, Padova University Hospital). Legend: **A** = Coronal image of low-dose CT; **B** = Sagittal image of low-dose CT; **C** = Axial image of low-dose CT; **D** = PET/CT fused image; **E** = Coronal image of PET; **F** = Sagittal image of PET; **G** = Axial image of PET; **H** = Maximum intensity Projection (MIP).

**Figure 5:**
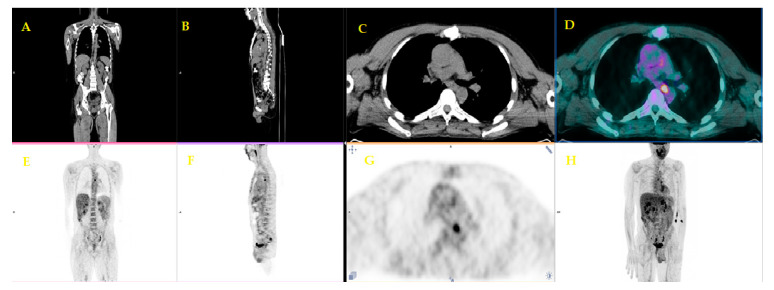
Lymph-nodal uptake suggestive of metastasis in the same patient (Images from Nuclear Medicine Unit, Padova University Hospital). Legend: **A** = Coronal image of low-dose CT; **B** = Sagittal image of low-dose CT; **C** = Axial image of low-dose CT; **D** = PET/CT fused image; **E** = Coronal image of PET; **F** = Sagittal image of PET; **G** = Axial image of PET; **H** = Maximum intensity Projection (MIP).

Figure 4 and Figure 5 show an example of a positive FDG PET/CT in a patient with a recent diagnosis of PDAC. FDG PET/CT was performed for the evaluation of potential distant metastases.

### 3.3. Clinical Management

The change of clinical management, provided by the imaging-adjusted impact, is probably the most important issue for PET imaging in PDAC [28]. FDG PET/CT may avoid invasive surgical procedures, showing supplementary metastatic sites in about 10% of cases and saving $1066 per patient [67]. Ghaneh et al. set the first multicenter, prospective, large-scale study (n = 261 patients) that aimed to estimate the added value concerning the cost-effectiveness of functional imaging to the standard diagnostic workup of PDAC [31]. For the diagnosis, CT had a specificity of 70.6% and a sensitivity of 88.5%, while FDG PET/CT had a specificity of 75.8% and a sensitivity of 92.7%. Namely, FDG PET/CT has proved useful in modifying the staging of PDAC for 10% of cases, changing the decision making in about 50% of cases and sparing non-useful surgery in 20% of cases. Further, Ergul et al. showed that FDG PET/CT has a potential role of changing the therapy planning in 30% of their cohort (n = 33), upstaging nine of them (detecting distant metastases in liver, lung, brain, mesentery and adrenal gland not seen with other imaging techniques) and downstaging one patient (spleen lesion described as a metastatic mass by ceCT and MRI) [16]. Additionally, Nishiyama et al. reported that FDG PET/CT adds information that is able to change the clinical initial staging in 12% of patients with a switch of the therapy [32].

During the restaging, Sperti et al. demonstrated that FDG PET/CT significantly influenced the therapeutic management in 44% of patients [68], while Albano et al. demonstrated influence in about 30% of cases [33]. Finally, Burge et al. demonstrated that PET/CT was able to avoid potential risks and futile surgery in 16% of patients with advanced pancreatic cancer [34]. However, it was not able to predict locally unresectable disease and demonstrated the inability to accurately define tumor extent relative to the surrounding tissues. This latter limitation would be overpassed by PET/MRI, as reported by Yeh et al. [69].

### 3.4. Tumor Recurrence

After resection, recurrences are frequent within two years, mainly in the post-operative bed, liver and peritoneum [35]. Each site of recurrence is associated with a different prognosis. In this context, FDG PET/CT can be considered a complementary imaging modality, useful in cases of mismatch between tumor marker increase and negative/equivocal ceCT findings [70]. Nishiyama et al. argued that FDG PET/CT has a complementary role compared to CT in detecting distant metastases in recurrent PDAC, with significantly higher sensitivity than that of CT (82% vs. 64%), while specificity was similar (98% vs. 98%) [32]. In the retrospective study by Sperti et al. PET was able to detect non-locoregional and extra-abdominal recurrences earlier than CT (especially in those patients with a pre-operative SUVmax > 6.0 at baseline PET), with a diagnostic accuracy of 96% and 57%, respectively [68]. Additionally, Albano et al. confirmed that a SUVmax value equal to 6 was accurate enough for the diagnosis of recurrent disease [33]. Furthermore, the authors reported that FDG PET/CT performed well in the restaging of PDAC, showing further metastasis in 10% of patients (5/52) and excluding suspicious foci in 21% of patients (11/52). In this clinical contest, FDG PET/CT had high sensitivity and specificity (about 85%) [33]. However, CT and MRI are superior to PET in liver metastasis assessment (92% vs. 42%), as reported by Ruf et al. [70]. Rayamajhi et al. demonstrated that FDG PET/CT represent a helpful tool in the case of doubtful ceCT and CA 19-9 normal [71]. In the differential diagnosis of recurrence, El Kholi et al. showed that benign and malignant lesions presented similar SUVmaxE (early image) but remarkably different SUVmaxD (delayed image) values; in fact, malignant lesions had more increased values (mean 8.6 ± 2.7) compared to benign ones (mean 3.3 ± 1.4). They also found that an SUVmaxD value of 4.9 was the most accurate (94.1%) in predicting malignant lesions (sensitivity 95.8%). Moreover, PPV and NPV were 90.0% and 95.8%, respectively [72]. However, the real advantage of PET imaging, in recurrent pancreatic cancer, is the ability to distinguish treatment-related fibrosis and inflammation from residual or progressive tumors, as clearly stated by Javery et al. in their 49 patient cohort [73].

**Figure 6:**
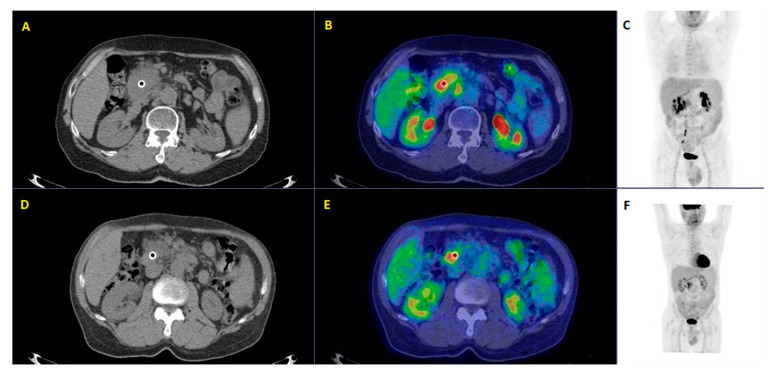
A fifty-four-year-old patient affected by local invasive ductal adenocarcinoma underwent restaging ^18^F-FDG PET/CT before and after chemotherapy. ^18^F-FDG PET/CT after 4 cycles of FOLFIRINOX (**A**–**C**) compared to the same examination before chemotherapy (**D**–**F**) revealed increasing metabolic activity of the pancreatic lesion (SUVmax = 14 vs. 10) and the appearance of a new locoregional lymph-nodal uptake in the surrounding fat (SUVmax = 7), demonstrating the lack of therapy response with the subsequent modification of therapy management. (Images from Nuclear Medicine Unit, Fondazione Istituto G.Giglio of Cefalù, Italy). Legend: **A** = Axial image of low-dose CT after chemotherapy; **B** = PET/CT fused image after chemotherapy; **C** = Maximum intensity Projection (MIP) after chemotherapy; **D** = Axial image of low-dose CT before chemotherapy; **E** = PET/CT fused image before chemotherapy; **F** = Maximum intensity Projection (MIP) before chemotherapy.

Figure 6 shows an example of a patient who underwent FDG PET/CT before and after chemotherapy. The second FDG PET/CT scan was made for the assessment of response to therapy and for the restaging of disease.

### 3.5. Treatment Response Assessment and Radiotherapy Planning

A complete resection represents the only potential cure; however, other treatment modalities, such as chemotherapy and radiation therapy, alone or in association, can help both in neoadjuvant and in adjuvant settings [69]. Chang et al. observed that, in a large study-cohort of metastatic patients (n = 388), FDG PET/CT resulted in switching to systemic treatments, thus avoiding a futile surgical approach [36]; namely, the authors found that the presence of a low SUVmax in the primary tumor and the reduction of SUVmax > 60% after therapy was associated with a better overall survival (OS) and progression-free survival (PFS). Similar results were also reported by Kurahara et al. and Nasr Shaban et al. [37,38]. The reduction of SUVmax before and after chemotherapy, in terms of percentage, has been extensively reported by several authors, such as Chang et al. [36], while others reported the utility of employing the European Organization for Research and Treatment of Cancer (EORTC) criteria for the definition of metabolic response to therapy [39]. Choi et al. reported that, in 20 patients with locally advanced pancreatic cancer undergoing neoadjuvant induction chemotherapy followed by concurrent chemoradiotherapy (chemo-RT), the 1-year survival rate for the PET responders was 87% and for the PET non-responders it was 28% [40]. Therefore, following the authors, FDG PET/CT may be used to aid patients who could have complete surgical resection as well as prognosticate patients’ survival. In the evaluation of response to therapy, however, data concerning new therapies for the treatment of pancreatic cancer, such as hormonal therapy, are not promising with FDG PET/CT. Eckel et al. enrolled 19 patient candidates to a 28-day course of SR 27897B, a highly selective non-peptide cholecystokinin receptor antagonist [41]. Imaging studies, including FDG PET/CT and MRI, were performed at baseline and on days 14 and 28. No significant changes in FDG uptake by the primary tumors were observed. SR 27897B, when used alone at the limited doses employed, led neither to an impairment of tumor glucose metabolism nor to a reduction of tumor size in advanced pancreatic cancer. In conclusion, an unchanged FDG uptake cannot be used as a measure of disease stabilization [42]. Additionally, radiation therapy can be used during neoadjuvant treatments in association with chemotherapy or for the completeness of the surgical approach, as intraoperative radiotherapy (IORT). Some papers have been published regarding the testing of the utility of FDG PET/CT for monitoring the response to IORT or radiotherapy [42] or for guiding to a specific radiotherapy planning [43,44]. Higashi et al. found that the measurement of the SUV could evaluate the local response of pancreatic cancer after IORT earlier and more accurately than with CT [42]. Further, Kishi et al. found that, in 14 patients, the tumor volume was significantly larger when delineated using four dimensional (4D)-PET than with three dimensional (3D)-PET [43]. Therefore, the internal target volume (ITV) generated from 4D-PET can be used to improve the accuracy or reduce normal tissue. Parlak et al. published a study based on 30 patients with locally advanced pancreatic cancer, showing that patients with lower gross tumor volume (GTV) assessed by FDG PET/CT have a significantly better OS than those with larger GTV during systemic therapies [45]. Finally, Wilson et al. assessed the role of FDG PET/CT at baseline and six weeks post-chemo-RT in locally advanced pancreatic cancer [44]. The authors found that the volume derived from 40% of the SUVmax predicts the geographical location of residual metabolically active tumors post-CRT in most patients (about 90% of cases) [44].

### 3.6. Prognosis

The opportunity to estimate the prognosis before surgery could guide therapeutic choice appropriately. Choi et al. showed that SUVmax is a valid tool in the pre-surgical prognostic stratification because a value higher than 3.5 in the primary tumor was associated with a significantly shorter OS and PFS [46]. Moreover, both high SUVmax and scarcely differentiated tumor histology were independently poor prognostic factors. Similarly, Yamamoto et al. demonstrated that the preoperative SUVmax of the primary tumor may be useful for selecting treatment strategies by using a cut-off value of SUVmax ≥ 6.0 [47]. Namely, in their study, 49% of patients with SUVmax ≥ 6.0, presented early recurrences (OS = 18 months). On the contrary, early recurrence in patients with a SUVmax < 6 happened in only 5% of cases (OS = 37 months) [47].

Another study showed that patients who presented at baseline a SUVmax equal to or less than 3.65 in the primary tumor had significantly better survival than those with SUVmax > 3.65 (*p* < 0.001) [48]. Pergolini et al. reported that an SUV max ≥ 6 in the preoperative evaluation is independently related to a poor PFS after surgery, identifying, in combination with high level of tumor biomarkers such as Ca 19.9, a subgroup of patients who may benefit from a systemic approach with neoadjuvant treatment [49]. Smeets et al. demonstrated that an accurate respiratory gating on quantification of 4D PET-metrics in PDAC significantly influenced the SUV calculation, which had a direct impact on their correlation with the OS [50]. A study by Choi et al. focusing on the prognostic role of volume based-PET parameters, showed a longer OS for low SUVmax, TLG, or MTV values in primary tumor [51]. Moreover, they obtained that the TLG represented an independent prognostic factor for OS, while MTV and Ca19-9 levels before chemo-RT also represented an independent prognostic factor for PFS [51]. A study by Su et al. aimed to explore the relative thresholds of volume-based PET/CT parameters in predicting the prognosis of locally advanced pancreatic cancer treated by stereotactic body radiation therapy [52]; the results underlined that both MTV (40%) and TLG (40%) correlated with OS (MTV, *p* = 0.029; TLG, *p* = 0.045), and MTV (40%) correlated with PFS (*p* = 0.026). Additionally, the MTV (40%) showed a stronger prognostic power than the TLG (40%) as an independent indicator for OS (*p* = 0.012) [52]. Similar results were obtained by Ren et al. [53]; in fact, in their experience, the MTV and TLG were significantly associated with PFS and OS (*p* < 0.05); additionally, TLG, radiotherapy dose, and chemotherapy were independent prognostic indicators of PFS and OS. Huang-Xian et al. analyzed the samples of 122 patients with resectable PDAC, evaluating the predictive preoperative role of FDG PET/CT semi-quantitative parameters (SUVmax, MTV, and TLG) for OS and PFS based on the tumor burden [54]. Their results demonstrated an important relationship between metabolic parameters with tumor size and baseline serum Ca19-9 level; furthermore, they were independent predictive factors of outcome in patients with resectable PDAC.

More recently, Mohamed et al. proved that a cut-off of 55 for TLG can differentiate the patients’ median survival (18 vs. 5 months, respectively, *p* < 0.001) and that, on multivariate analysis, the TLG (*p* < 0.004) and the presence of distant metastasis (*p* < 0.001) emerged as independent prognostic factors [55]. Hyung-Yun et al. and Lee et al. showed similar results, adding the association with lymphovascular involvement [35,56]. Yong-il Kim et al. demonstrated that volumetric parameters represented independent prognostic factors and found that the heterogeneity index, obtained by a linear regression analysis of the MTV, can be an additional predictor of recurrence in patients with a surgically treated PDAC [57]. Further, Hyun et al. using a radiomics approach, observed that tumoral heterogeneity assessed in FDG PET/CT images was independently related to prognosis together with stage and serum Ca19-9 levels [58]. Additionally, Toyama et al. performed radiomics analysis in 161 patients with pancreatic cancer and revealed that, among the 42 features extracted, grey-level zone length matrix (GLZLM) grey-level non-uniformity (GLNU) was the only statistically significant PET parameter for predicting one-year survival, followed by TLG [59].

Lee et al. introduced a parameter named “SUVgluc” to correct the FDG uptake for the blood glucose level, evaluating the role of FDG PET/CT in the prognostic prediction after curative resection [60]. According to their results, the SUVgluc resulted significantly higher in the recurrence subset than in the non-recurrence one. ROC analysis revealed that a SUVgluc of 4.8 was the best cut-off value to predict metastases; lower and higher values were related to a nine-month PFS approximate of 72% and 23%, respectively [60]. Nakajo et al. examined, for the first time, the prognostic value of [^18^F]fluoro-3′-deoxythymidine [^18^F]-FLT PET/CT compared to FDG PET/CT in PDAC [61]. The authors found that FLT positively correlated with cell growth and TK-1 activity and could be similar to FDG PET/CT in revealing metastatic foci (except liver metastases), improving the prognostic assessment [61].

## 4. Discussion and Conclusions

PDAC remains a lethal disease with a poor outcome [4]. Nowadays, it is diagnosed through clinical evaluation and a sequence of imaging tools including CT, MRI, and EUS [74]. The long-term survival of patients with PDAC remains poor, mainly because of the resistance to treatments and undiagnosed lesions during surgical procedures; therefore, accurate oncological management of patients is mandatory. Although the routine use of FDG PET/CT is not well established, functional imaging can provide useful information and hold a relevant position in the whole management of PDAC. A relevant question regards the differential diagnosis between malignant and benign pancreatic lesions due to the differences in prognosis and treatment implications [19,20]. Furthermore, the sum of information provided by SUVmax in primary and in the lymph node metastasis and Ca 19.9 can help to identify patients who may benefit from neoadjuvant treatment [71]. It has been estimated that 100% of patients with a complete response to neoadjuvant therapy at FDG PET showed a complete surgical resection [74]. After resection, recurrences are frequent, mainly in the post-operative bed, liver and peritoneum. In this setting, FDG PET/CT plays the main role and a complementary position compared to CT but it has a lower accuracy in the detection of liver metastases [70]. Probably the combination of more than one imaging modality can help the clinicians to better understand the widespread recurrent disease.

The change of clinical management, provided by imaging-adjusted impact, is another important issue for FDG PET/CT imaging in PDAC. FDG PET/CT may determine the avoidance of unnecessary surgery by detecting additional metastatic disease, significantly influencing the therapeutic management in about 30% of cases. Moreover, PET metabolic semi-quantitative parameters such as SUV, TLG, and MTV proved to be independent prognostic factors for PDAC. Therefore, FDG PET/CT may also play an important role in risk stratification, giving clinicians the chance to evaluate prognostic factors and to adopt more effective therapies. 

From the analysis of the literature, it is undoubtedly clear that FDG PET/CT should be heavily embedded in clinical practice. Especially when considering initial staging and treatment planning, FDG PET/CT showed robust efficacy in the management of patients with PDAC. Although data on surgical and radiotherapeutic planning for monitoring the treatment response and recurrent disease and on cost-effectiveness are somewhat more limited and require further investigation, current data support a wider application of FDG PET/CT even in these settings. In this regard, previous experiences with other neoplasms characterized by a high degree of glucose consumption provides the rationale for wider applicability of FDG PET in clinical practice. Furthermore, FDG PET can play a major role in the concept of multimodality imaging, which has been proven to be critical in the diagnosis and management of pancreatic cancer [74].

However, some limitations of the present review should be acknowledged. Namely, the selected papers are mainly retrospective, featuring small patient populations. Indeed, more prospective, randomized trials are needed to provide robust evidence of the pivotal role of FDG PET/CT in the management of patients with PDAC. However, it should be noted that the quality assessment using the QUADAS-2 tool revealed that the majority of the selected papers fulfil adequate quality standards.

In conclusion, besides conventional anatomical imaging, such as CT and MRI, molecular imaging with FDG PET/CT can be used in all phase of disease but, considering the limited role at diagnosis for a low specificity and for limited results about the use in response to therapy assessment, this morpho-functional tool showed the potential best performances for preoperative staging, recurrence detection and prognosis estimation of PDAC. However, this diagnostic method still awaits a complete integration into the diagnostic workup and also requires recognition of its value in the clinical routine that can only be obtained through further prospective studies to confirm the cost-effectiveness based on the potential improvement of clinical management.

## Figures and Tables

**Table 1:** Main findings and summary of articles selected.

N	Authors	Year	Study Design	Number of Patients and Age (Mean ± DS or Range)	Disease Phase and Eventual Treatment	Findings
	**Diagnosis**					
1	Ergul et al. [16]	2013	Retrospective study	52 (63.4 ± 11.7)	Staging	Fluorodeoxyglucose (FDG) positron emission tomography (PET)/computed tomography (CT) seems to be useful, especially when applied with endoscopic ultrasound as first line diagnostic tools.
2	Zhang et al. [17]	2019	Retrospective study	111	Staging	FDG PET/CT and radiomics method help in non-invasive diagnosis of autoimmune pancreatitis, especially when biopsy is inconclusive.
3	Buchs et al. [18]	2010	Prospective study	45 (69 (22–82))	Staging	FDG PET/CT offers good sensitivity in the detection and assessment of pancreatic cancer, but at the price of a relatively low specificity
4	Kato et al. [19]	2012	Retrospective study	47 pancretic lesions (33 PDAC) (66 ± 8.6)	Staging 20 patients then operated	Limit of the standardized uptake value (SUV) max values in distinguishing between chronic pancreatitis and pancreatic ductal adenocarcinoma (PDAC), except for extreme values.
5	Hu et al. [20]	2013	Retrospective study	80 (57.3 ± 12.4)	Staging pancreatoduodenect-omy (30 patients), distal pancreatectomy (36 patients), total pancreatectomy (4 patients), proximal pancrea-tectomy (2 patients), and lesion resection (8 patients)	SUVmax of malignant tumors had a positive correlation with Ki-67, so helped in malignant lesions diagnosis.
	**Preoperative Staging**					
6	Myssayev et al. [21]	2014	Retrospective study	48 (68.2)	Preoperative Staging biopsy-proven pancreatic adenocarcinoma	TLG (total lesion glycolysis) and MTV (metabolic tumor volume) are superior to SUV-based parameters for predicting tumor aggressiveness but are not directly related to vascular infiltration status and are not helpful alone for taking decision to perform surgery.
7	Strobel et al. [22]	2015	Retrospective study	50 (64.3)	Preoperative staging biopsy-proven pancreatic adenocarcinoma	The one-stop-shop imaging approach is superior to unenhanced PET/CT in defining the resectability of PDAC, improving the detection of distant metastasis.
8	Asagi et al. [23]	2013	Prospective study	108	Preoperative staging in advanced disease	PET/contrast enhanced computed tomography (ceCT) imaging can provide useful information in the clinical management of pancreatic cancer.
9	Picchio et al. [24]	2012	Prospective study	42	Patients selection for helical tomotherapy with concurrent chemotherapy	PET/CT influenced the treatment strategy by detecting distant metastases not documented by CT, thus accurately selecting patients for hormonal-chemotherapy after induction chemotherapy
10	Casneuf et al. [25]	2007	Retrospective study	46	Diagnosis, staging, and restaging of pancreatic lesions	The accuracy rate of PET/CT (91.2%) for diagnosis of primary pancreatic lesions is higher compared to CT (88.2%) and PET alone (82.3%). Additionally, for locoregional staging, PET/CT has a higher accuracy rate (85.3%) compared to CT (83.8%) and PET (79.4%).
11	Lemke et al. [26]	2004	Retrospective study	104	Preoperative staging	The image fusion (PET/ceCT) permits a more accurate assessment of the resection criteria, also improving the correct anatomic localization of small lesions
12	Yoneyama et al. [27]	2014	Retrospective study	95 (67 (36–83))	Staging	The magnitude of diagnostic accuracy of PET/contrast enhanced CT in the detection of distant metastasis, lymph node metastasis, and peritoneal dissemination remains still unclear
13	Wang et al. [28]	2019	Retrospective study	160 (66)	Preoperative staging surgical resection within 1 week after the 18F-FDG PET/CT scan	Either CT or PET are limited in evaluations of node metastasis. The best SUVmax and CA 19-9 cut-off values for predicting lymph node micrometastases is 7.05 and 240.55 U/mL, respectively.
14	Kim et al. [29]	2018	Retrospective study	85 (69 (41–89))	Preoperative staging	SUV of the lymph nodes (SUVLN) seems to be a more significant prognostic factor in pancreatic cancer than the primary tumor’s SUV
15	Kaida et al. [30]	2016	Retrospective study	53 (68 (40–81))	Preoperative staging	FDG uptake may predict the levels of endothelial growth factor receptor (EGFR) and p70S6 expressions, whilst mTOR did not correlate with FDG uptake
	**Tumor Recurrence**					
16	Ghaneh et al. [31]	2018	Prospective study	589	Whole management	FDG PET/CT, in addition to standard diagnostic work-up of PDAC, correctly changed the staging of PDAC in 10% of cases, influenced the planned management in 45%, avoided un-useful resection in 20% of patients scheduled for surgery, and got a limited role in chronic pancreatitis.
17	Nishiyama et al. [32]	2005	Retrospective study	42 (65.8 (33–93))	Restaging	FDG PET/CT adds information that was able to change the clinical initial staging in 11.9% of patients with a change of the therapy
18	Albano et al. [33]	2018	Retrospective study	52 (59 (42–78))	Restaging 28 surgery, 12 neoadjuvant chemotherapy+surgery+radiotherapy, s6 neoadjuvant chemotherapy+surgery, and 6 chemotherapy	PET/CT has a high diagnostic accuracy in the restaging process and significantly influences the therapeutic management in ∼30% of cases.
19	Burge et al. [34]	2015	Prospective study	56 (64 (35–84))	Staging and evaluation of impact of PET/CT on management	PET/CT was able to avoid potential risks and futile surgery in 16% of patients with advanced pancreatic cancer. However, it was not able to predict locally unresectable disease
20	Hyung-Jun et al. [35]	2016	Retrospective study	51	Staging Surgery and adjuvant treatment in all patients (concurrent chemo- radiotheraphy in 41, chemotheraphy in 9, radiotheraphy in 1) after scan	MTV and TLG are associated with the presence of lymphovascular invasion.
	**Therapy Assessment**					
21	Chang et al. [36]	2014	Retrospective study	388	Staging and post-Therapy assessment	PET/CT resulted in the ability to switch to systemic treatments, avoiding a futile surgical approach. Namely, the authors found that the presence of a low SUVmax in the primary tumor and the reduction of SUVmax > 60% after therapy was associated with a better overall survival (OS) and progression-free survival (PFS)
22	Kurahara et al. [37]	2018	Retrospective study	125	Pretreatment evaluation	FDG PET SUVmax was significantly associated with the therapeutic response to chemoradiotheraphy (CRT) in PDAC patients
23	Nasr Shaban et al. [38]	2015	Retrospective study	20 (60.25 (57–74))	Post-Therapy assessment	Combined FDG PET/CT significantly improves the sensitivity and specificity of isolated CT for depicting pancreatic tumors and distant metastases and can monitor response to treatment, distinguishing fibrosis from residual/recurrence
24	Korn et al. [39]	2017	Prospective study	52	Early PET imaging in patients with metastatic pancreatic adenocarcinoma (mPC) treated with nab-paclitaxel plus gemcitabine	PET effectively measured changes in tumor metabolic activity at 6 and 12 weeks. These results support the antitumor activity of nab-paclitaxel 125 mg/m^2^ plus gemcitabine 1000 mg/m^2^ for treating mPC and the utility of PET for measuring treatment response. Treatment response by PET analysis may be considered when evaluating investigational agents in mPC.
25	Choi et al. [40]	2010	Retrospective study	20	Early treatment response	FDG-PET-CT has an important role in defining the gross tumor volume (GTV) size in predicting outcomes of locally advanced pancreatic cancer (LAPC)
26	Eckel et al. [41]	2002	Prospective study	19 (62 (43–76))	PET at baseline and on days 14 and 28 in monitoring hormonal therapy using a highly selective, non-peptide cholecystokinin (CCK) receptor antagonist	No significant changes in FDG uptake by the primary tumors were observed. SR 27897B, when used alone at the limited doses employed, led neither to an impairment of tumor glucose metabolism nor to a reduction of tumor size in advanced pancreatic cancer. An unchanged FDG uptake cannot be used as a measure of disease stabilization.
27	Higashi et al. [42]	1999	Retrospective study	14	PET before (n = 12) and after IORT (0.7–11.9 mo, n = 14)	FDG PET was useful in monitoring patients after intraoperative radiotherapy (IORT), because the decrease of metabolism in pancreatic tumors could be detected earlier than the decrease in tumor size
28	Kishi et al. [43]	2016	Retrospective study	14	Four dimensional (4D)-PET in pancreatic cancer radiotherapy treatment planning	Tumor volume was significantly larger when delineated using 4D-PET than three dimensional (3D)-PET
29	Wilson et al. [44]		Retrospective study	17 (65 (45–74))	Staging	Low pre-CRT FDG-avidity related to less likely development of metastatic disease.
30	Parlak et al. [45]	2012	Prospective study	30 (57 (39–68))	FDG-PET-CT based radiotherapy planning	Patients with lower gross tumor volume (GTV) assessed by FDG PET/CT have a significantly better OS than those with larger GTV during systemic therapies
	**Prognosis**					
31	Choi et al. [46]	2013	Retrospective study	64 (63.5 (45–30))	Restaging 34 (53.1%) underwent pylorus-preserving pancreatoduodenectomy, 18 (28.1%) distal pancreatectomy, 10 (15.6%) pancreatoduodenectomy, and two (3.1) total pancreatectomy. 40 patients had adjuvant treatment, 28 had chemotherapy, and 12 had chemoradiotherapy.	High SUVmax is an independent poor prognostic factor and may play an important role in risk stratification and treatment planning prior to undertaking surgical resection.
32	Yamamoto et al. [47]	2014	Retrospective study	128 (67)	Staging pancreaticadenocarcinoma that preoperatively underwent FDG-PETexaminations	SUVmax cut off value 6.0 may be useful for selecting treatment strategies.
33	Sperti [48]	2020	Retrospective study	144 (66.3)	Staging	The SUVmax calculated with 18-FDG-PET/CT is an important prognostic factor for patients with pancreatic cancer and may be useful in decisions concerning patients’ therapeutic management.
34	Pergolini et al. [49]	2017	Retrospective study	46 (67)	Staging All pancreaticoduodenectomy	Preoperative SUVmax ≥ 6 is an independent predictor of poor disease-free survival (DFS) and disease specific survival (DSS) after surgery, identifying, in combination with other biomarkers of aggressiveness like CA 19.9, a subgroup that can benefit from a systemic approach with neoadjuvant treatment.
35	Smeets [50]	2019	Retrospective study	69 (66 (40–82))	Staging	Amplitude-based optimal respiratory gating (ORG) on quantification of PET-derived image features in PDAC has a significant impact on all measured metabolic parameters.
36	Choi et al. [51]	2014	Retrospective study	60 (64.7)	Staging chemoradiation therapy after scan	The disease control rate (DCR) is significantly higher in patients with low SUVmax, MTV, or TLG, and has a strong correlation with longer survival times.
37	Su et al. [52]	2020	Prospective study	35 (67.2 (45–84))	Pre-SBRT Staging	MTV (40%) was the optimal prognosticator among the relative thresholds of SUVmax for tumour delineation on PET/CT for LAPHC patients receiving stereotactic body radiation therapy (SBRT).
38	Ren et al. [53]	2020	Retrospective study	73 38 pts ≤ 68y 35 > 68y	Restaging	TLG was found to be the independent prognostic factor of OS, and PFS TLG was found to be the independent prognostic factor of OS and PFS
39	Huang-Xian et al. [54]	2014	Retrospective study	122 (62 (35–84))	Staging Radical pancreatectomy after scan	Preoperative MTV and TLG values are significantly associated with baseline serum CA19-9 level and tumor size.
40	Mohamed et al. [55]	2020	Retrospective study	89 (69 (44–85))	Staging	Tumor TLG offer an independent prognostic value in both potentially operable and metastatic disease settings
41	Lee et al. [56]	2014	Retrospective study	87 (61 ± 10)	Staging	MTV and TLG measured on preoperative FDG PET/CT are independent and significant prognostic factors for predicting overall survival and recurrence free survival.
42	Yong-il Kim et al. [57]	2017	Retrospective study	93 (64.2 ± 9.1)	Staging Surgery (+Adjuvantherapy in 76/93) after scan	Heterogeneity index could be a predictor of recurrence in surgically resected PDAC. Additionally, volumetric parameters, as well as venous invasion, are independent prognostic parameters.
43	Hyun et al. [58]	2016	Retrospective study	137 (63 (36–87))	Staging 80 Curative surgery with or without adjuvant therapy, 14 concurrent chemoradiotherapy, 17 Chemotherapy alone, and 26 Best supportive care	Tumoral heterogeneity of ^18^F-FDG PET/CT uptake by texture analysis is an independent predictor of survival along with tumor stage and serum Ca 19-9 level.
44	Toyama et al. [59]	2020	Retrospective study	161	Staging	Among the 42 features extracted, gray-level zone length matrix (GLZLM) gray-level non-uniformity (GLNU) was the only statistically significant PET parameter for predicting 1-year survival, followed by TLG
45	Lee et al. [60]	2011	Retrospective study	43 (62 (31–79))	Staging 23 pancreatoduodenectomy, 15 distal pancreatectomy, and 5 total pancreatectomy after scan Adjuvant radiotherapy in 29 patients within 1 month after the operation	Introduction of SUV correction for the blood glucose level calculated as SUVgluc (SUVmax x blood glucose level/100 mg/dL). Values are significantly higher in the recurrence group than in the non-recurrence group.
46	Nakajo et al. [61]	2016	Prospective study	15 (69 ± 12)	Staging 4 pancreaticoduodenectomy (within 2 adjuvant chemotherapy)	Prognostic value of FLT-PET/CT is potentially equivalent to ^18^F- FDG PET/CT for detecting primary and metastatic PDAC, except liver metastasis.
